# Team-Based Integrated Knowledge Translation for Enhancing Quality of Life in Long-term Care Settings: A Multi-method, Multi-sectoral Research Design

**DOI:** 10.15171/ijhpm.2019.123

**Published:** 2019-12-01

**Authors:** Janice Keefe, Mary Jean Hande, Katie Aubrecht, Tamara Daly, Denise Cloutier, Deanne Taylor, Matthias Hoben, Kelli Stajduhar, Heather Cook, Ivy Lynn Bourgeault, Leah MacDonald, Carole A. Estabrooks

**Affiliations:** ^1^Department of Family Studies and Gerontology and Nova Scotia Centre on Aging, Mount Saint Vincent University, Halifax, NS, Canada.; ^2^Mount Saint Vincent University, Halifax, NS, Canada.; ^3^Department of Sociology, Saint Francis Xavier University, Antigonish, NS, Canada.; ^4^Faculty of Health and York University Centre for Aging Research and Education, York University, Toronto, ON, Canada.; ^5^Department of Geography, University of Victoria, Victoria, BC, Canada.; ^6^Interior Health Authority British Columbia, Kelowna, BC, Canada.; ^7^Faculty of Nursing, University of Alberta, Edmonton, AB, Canada.; ^8^Institute on Aging and Lifelong Health and School of Nursing, University of Victoria, Victoria, BC, Canada.; ^9^Office of the Seniors Advocate, Victoria, BC, Canada.; ^10^Tefler School of Management, University of Ottawa, Ottawa, ON, Canada.; ^11^Vancouver Island Health Authority, Victoria, BC, Canada.; ^12^Faculty of Nursing and School of Public Health, University of Alberta, Edmonton, AB, Canada.

**Keywords:** Late Life, Long-term Care, Integrated Knowledge Translation, Quality of Life, Canada

## Abstract

Multi-sectoral, interdisciplinary health research is increasingly recognizing integrated knowledge translation (iKT) as essential. It is characterized by diverse research partnerships, and iterative knowledge engagement, translation processes and democratized knowledge production. This paper reviews the methodological complexity and decision-making of a large iKT project called Seniors - Adding Life to Years (SALTY), designed to generate evidence to improve late life in long-term care (LTC) settings across Canada. We discuss our approach to iKT by reviewing iterative processes of team development and knowledge engagement within the LTC sector. We conclude with a brief discussion of the important opportunities, challenges, and implications these processes have for LTC research, and the sector more broadly.

## Introduction

A pan-Canadian, multi-disciplinary research project, “Seniors – Adding Life to Years (SALTY)” represents a team-based integrated knowledge translation (iKT) approach to address complex and pressing challenges facing long-term care (LTC). In this paper, we describe our iKT approach, which built on pre-existing, cross-jurisdictional and cross-sectoral research relationships to explore pressing questions about life in LTC. After reviewing themes from relevant literature on knowledge translation (KT), we outline our key processes for building a diverse “team of teams” comprised of LTC representatives and stakeholders – ranging from policy-makers, clinicians, care aides, family members and residents. The iKT mechanisms we have built and fine-tuned support their continued engagement in the research process. We conclude with a preliminary assessment of the effectiveness of our iKT approach and implications both for LTC and the field of KT in health research more generally.

## Knowledge Translation

Social, political and economic changes over the past 30 years have resulted in a shift in the social contract between science and society. Now science must demonstrate itself to be socially relevant (not just rigorous and reliable), and public research dollars are increasingly contingent on demonstrating social and economic “returns on investment.”^1^ This climate has fostered the development of KT theories, methods and measures that inform a growing body of research practices to address problematic gaps in translating “knowledge to action” in the health and policy sector. In Canada, major funding bodies, such as the Canadian Institutes of Health Research (CIHR) play a significant role in guiding KT science and practice. The CIHR^2^ defines KT as a dynamic and iterative process that includes synthesis, dissemination, exchange and ethically-sound application of knowledge to improve the health of Canadians, provide more effective health services and products and strengthen the healthcare system. This process takes place within a complex system of interactions between researchers and knowledge users which may vary in intensity, complexity and level of engagement depending on the nature of the research and the findings as well as the needs of the particular knowledge user.

“End-of-grant” and “integrated” KT processes are also distinguished. End-of-grant KT models map the translation of findings once research is deemed “complete.” iKT “applies the principles of KT to the entire research process.”^3^ This involves a greater emphasis on engagement (rather than simply transfer) activities with knowledge users who are positioned as research “partners” throughout each stage of the research process. Bowen and Graham^4^ note that this latter focus on knowledge engagement marks a divergence from traditional scientific research and draws instead on social science methodology that tends to focus on process, change management, and challenging the status quo in health organizations.

CIHR notes that iKT shares many similarities with research approaches—such as “collaborative research, participatory action research, community-based participatory research, co-production of knowledge or Mode 2 research”^3[1] ^—that have grown in popularity over the last 60 years. In a healthcare context, iKT has broadened the scope of research partnerships from traditional experts (ie, clinicians, policy-makers) to frontline care workers, patients, residents, and their families. These shifts have the *potential* to transform research partnerships^5,6^ and challenge notions of what constitutes expertise^7^ and even knowledge itself through democratization of knowledge processes.^8,9^ In some iKT projects, knowledge democratization is achieved through including and prioritizing the perspectives of knowledge users, broadening definitions of “valid knowledge,” flattening hierarchical research governance structures through critical partnership models, and “loosen[ing] up the restrictive distinctions and ways of relating (eg, distinguishing between practitioners/researchers, knowers/nonknowers, and knowledge/action).”^8^

Yet, in reflecting on the transformative potential of iKT research partnerships, Gagliardi et al^10^ argue that an “absolute partnership is not attainable” as power dynamics and the rewards and disadvantages for each of the respective partners are uneven. Many researchers argue that the democratization of research and the expansion of what constitutes knowledge involve a more profound shift away from traditional, linear and biomedical approaches to research to a more iterative, reflexive engagement process. For example, Carayannis et al^11^ promote a move beyond knowledge application and innovation (also known as “Mode 2” research) to “Mode 3” research that intentionally diversifies the organizational context in which knowledge is produced. This requires research partners move away from a “first then” to an “as well as” or a “parallelized” relationship between knowledge production and implementation or innovation. Hartrick Doane et al^8^ argue that iKT researchers would do well to move away entirely from KT’s preoccupation with the “knowledge-*to* -action gap,” and instead engage directly *with* the gap itself, a move that requires thinking in terms of “knowledge-*as* -action,” where iKT itself is the gap. Greenhalgh and Wieringa’s^12^ exploration of various approaches to facilitating “macro-level knowledge partnerships between researchers, practitioners, policy-makers and commercial interests,” exemplifies a collaborative and relationship-centred approach to knowledge-as-action that has been influential in SALTY’s iKT approach.

## Adding Life to Years in Long-term Care

With increasingly complex care needs and decreasing lengths of stay in LTC,^13^ there is a pressing need to improve quality of life and address perennial problems in the sector. Frequently cited challenges include lack of timely and quality care, lack of evidence to inform clinical decision-making, inadequate resource allocation and staffing levels, and overall challenges with quality of care.^14^ One major hurdle to institutional change is the complexity of LTC. LTC facilities serve as workplaces, homes, health institutions and makeshift sites for accessing and coordinating a variety of public and private services with sometimes contradictory or uneven regulation.

Improving the quality of life in LTC facilities thus requires change on many fronts and in many jurisdictions. Healthcare policy, improvements in quality measures and monitoring, working conditions for staff, prioritizing financial investments, and broad socio-cultural shifts are among the areas where change is necessary.^15,16^ The last decade has seen [re-]invigorated attention on LTC, with new instruments and measures in Canada like the Resident Assessment Instrument – Minimum Data Set (RAI-MDS) 2.0^ [2]^ to inform our understanding of the population in LTC, and their care needs.^14^ However, critical challenges remain with how to ensure data quality, contextualize quantitative data, and determine how best to utilize the data to make system change,^18^ as well as with how to capture the complexities of these challenges.

The SALTY project emerged as an effort to bring together Canada’s considerable research and policy expertise to address these complex and pressing challenges. In 2015, a large CIHR funding competition on “Late Life Issues” helped consolidate these efforts into a research proposal with the central goal of supporting measurable contributions to improving the clinical and social experience of older adults during late life in LTC. SALTY has three guiding objectives: (1) Describe relationships between quality of care, work, life, and death; (2) Articulate promising palliative and end of life practices in LTC; and (3) Describe opportunities/limitations and directions for end of life measures to monitor quality of care.

**Figure 1:**
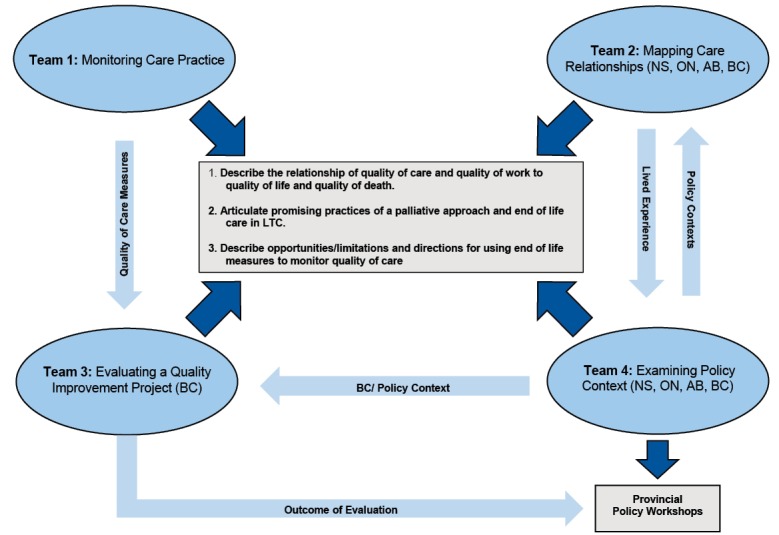
Interdependence Across SALTY Research Teams. Abbreviations: SALTY, Seniors - Adding Life to Years; LTC, long-term care; NS, Nova Scotia; ON, Ontario; AB, Alberta; BC, British Columbia.

**Figure 2:**
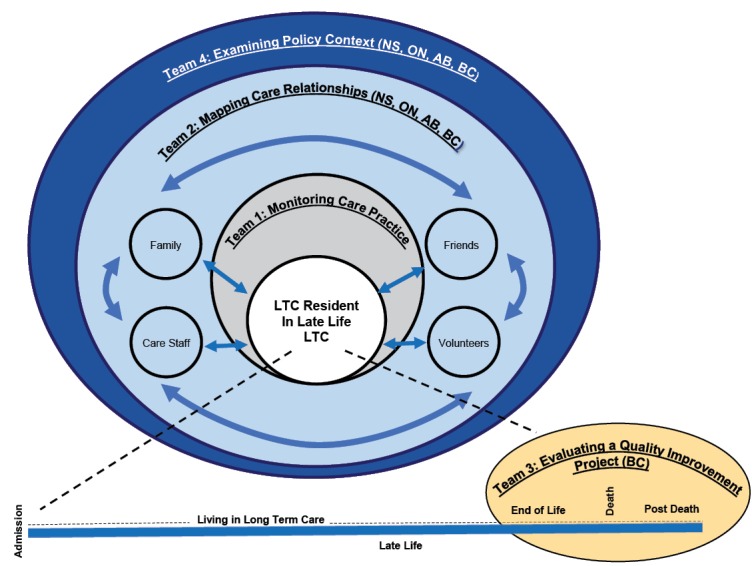
SALTY Research Teams. Abbreviations: SALTY, Seniors - Adding Life to Years; LTC, long-term care; NS, Nova Scotia; ON, Ontario; AB, Alberta; BC, British Columbia.

SALTY became a “team of teams” as academic researchers formed four multi-disciplinary research “teams,” each building on several years of collaboration involving at least five large research initiatives^ [3]^ in four Canadian jurisdictions: Alberta (AB), British Columbia (BC), Nova Scotia (NS), and Ontario (ON). Figure 1 shows how each team aims to integrate findings to contribute to SALTY’s central objectives. Team 1 uses RAI-MDS 2.0 data to develop longitudinal measures for burdensome symptoms and potentially inappropriate practices that have been selected as being highest priority at the end of life, by LTC residents, family and decision-makers. These measures will inform an understanding of end of life experiences in LTC and allow the implementation and evaluation of interventions to improve quality of care and by extension, quality of life. Team 2 uses a rapid site switching qualitative methodology^19^ to investigate promising relational approaches to care in late life. Through ethnographic case studies, this team reveals how quality of death and quality of life are inextricable from conditions of work for LTC residents, family and friends, volunteers, and paid staff. Team 3 evaluates a quality improvement implementation project to integrate a palliative approach to care into LTC; and to inform development of other Canadian LTC models. Finally, Team 4 considers how the regulatory environment across four provincial jurisdictions helps to enhance or limit quality of life in LTC. Using the policy lenses of LTC residents, families, staff and volunteers, these analyses contextualize the findings in Teams 1-3^ [4] ^. Figure 2 shows how each team provides research insights of varying scope and scale that can be triangulated^ [5]^ to strengthen the overall investigation of LTC residents’ experiences of late life.

## Democratizing Knowledge

**Figure 3:**
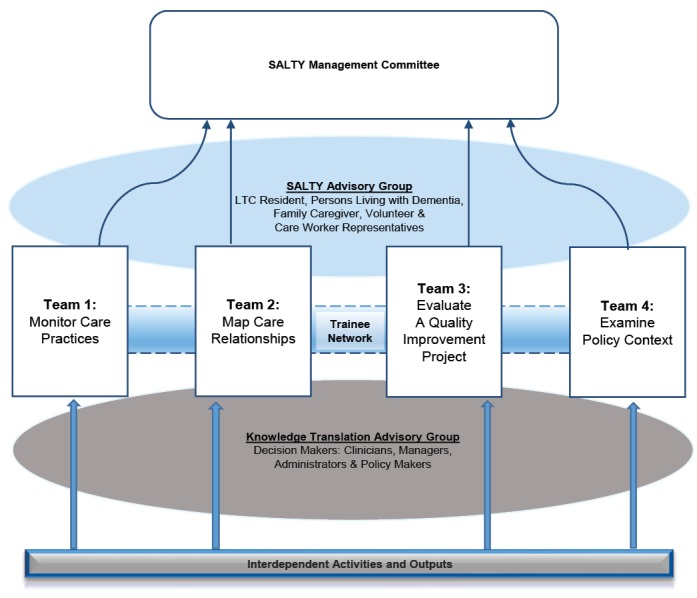
Governance/Partnership Model. Abbreviations: SALTY, Seniors - Adding Life to Years; LTC, long-term care.

Each team is linked by its interest in core concepts of quality of care and quality of life, which are often defined and operationalized differently within and across policy, practice, research, facilities and jurisdictional levels. Following CIHR’s iKT guidelines, the SALTY research team incorporates representation across multiple levels and jurisdictions across the Canadian LTC sector to ensure effective research integration, communication, representative decision-making, knowledge engagement and implementation at every stage of the research process. Figure 3 depicts SALTY’s governance across (1) research teams; (2) a cross-team trainee network of research assistants, graduate students and postdoctoral fellows; (3) a KT Advisory Group comprised of decision-makers, clinicians, managers, administrators, and policy-makers; and (4) a SALTY Advisory Group representing LTC residents, people with dementia, family members, care aides, and volunteers, recruited from pre-existing research projects and relationships. This latter Advisory Group represents those partners rarely included in LTC research, yet it was determined in the very early stages of the project, that their unique perspectives were invaluable. Team and trainee “Co-leads,” and advisory group chairs were established to foster interdisciplinary learning and KT and meet regularly as a Management Committee. Some team members play multiple roles on the project. For example, one member is a knowledge user (and chaired the KT Advisory Group), a trainee (co-leading the Trainee Network) and a co-lead on one of the research teams. Each of these mechanisms enhanced iKT through ongoing cross-pollination and engagement across a wide variety of research partners and research findings.

Overall, the project incorporated five iKT principles: (1) multi-jurisdictional and multi-level groups contribute to research design, and knowledge discovery; (2) diverse academic and knowledge user members help democratize knowledge production and balance perspectives to ensure robust findings; (3) adequate time and frequency of meetings allows for sufficiently thorough conversation and thoughtful decision-making across a wide spectrum of audiences, frameworks and skillsets; (4) in-person and videoconference meetings with members of all groups enables sharing of and reflection about ideas and findings; and (5) guiding policies for healthy communication and conflict resolution among team members and partners in all aspects of SALTY, including resource allocation, team structure, and team values. While these principles were initially guided by specifications from the CIHR grant competition, additional emphasis was placed on structures and team-building processes that aimed to further democratize knowledge production by forefronting marginalized voices, namely families, residents, people with dementia, volunteers and low-wage care workers.

## Discussion and Conclusion

This iKT approach was not without challenges. First, the size of the team and geographical distances among team members limited the number of face-to-face meetings that were possible both at the team and whole project levels. While face-to-face meetings often facilated the best forms of engagement, videoconferencing technology helped bring people together and bridge distances. Team members also worked together during fieldwork and met at knowledge dissemination events. Embedding team meetings within research and dissemination provided a way to practice stewartship, an integral part of the iKT approach. Second, significant time and attention was invested to support active and meaningful participation of non-academic team members, including clinicians, government and non-government decision-makers and advisory group members. Research teams routinely shared findings with the advisory groups for feedback and direction, and these are also involved in team meeting planning. The disciplinary, methodological and epistemological diversity of the SALTY team that supported the novelty of the SALTY program also required time and space to work through collectively as a team. A key way this was negotiated early in the program’s work was through a shared acknowledgement that consensus is not always possible, that dissenusus can be generative, and that “creative tensions” are a crucial and valuable aspect of the iKT research process. Involving the non-traditional team members during the project development and facilitating open discussion of how disciplines affect our perspection of results were critical to authentic participation and the respect for different points of view.

SALTY members characterize their “networked” iKT approach as one that builds on pre-existing relationships across the LTC sector, integrates research findings across distinct research teams and contextualizes knowledge from various scales, representatives and stakeholders in every stage of the research process. As a “team of teams,” articulating overarching themes across research teams with unique goals and epistemological commitments, was a challenge. However, implementing a governance structure aimed at continual integration and cross-pollination helped us work against tendencies to silo research within particular teams or sectors. Further, incorporating pre-existing research relationships, a diversity of stakeholders and reflexively attending to dynamics of underrepresentation, particularly in the SALTY Advisory Group, helped contextualize the various findings across each research team by presenting diverse perspectives and interpretations thus enhancing their relevance.

SALTY’s iKT approach also provided focus and leverage for a process that was already in motion. Rather than engage in a linear knowledge-*to* -action research model, SALTY’s dynamic iKT approach is perhaps better characterized as knowledge-*as* -action^8^ where the research team aimed to approach processes, outputs and outcomes as interconnected and iterative. It marks a shift towards the contextualized, democratized, relationship- and process-oriented iKT urgently needed in LTC and an approach for researchers and knowledge users considering an iKT approach to addressing complex social challenges.

## Acknowledgements

The authors acknowledge the SALTY team for its contributions to this study. This research is funded through a Late Life Issues grant from the CIHR [145401] in partnership with the Michael Smith Foundation for Health Research, Research Nova Scotia Foundation and the Alzheimer Society of Canada. We also acknowledge Pamela Fancey and Paula Richardson for their specific assistance reviewing and preparing this manuscript for submission.

## Ethical issues

Not applicable.

## Competing interests

Authors declare that they have no competing interests.

## Authors’ contributions

Conception and design: JK, MJH, KA, TD, DC, DT, KS, ILB, and CAE; Drafting of the manuscript: MJH; Critical revision of the manuscript for important intellectual content: JK, KA, TD, DC, DT, MH, KS, HC, and CAE; Obtaining funding: JK, KA, TD, DC, DT, MH, KS, HC, ILB, LM, CAE; Supervision: JK.

## Authors’ affiliations

^1^Department of Family Studies and Gerontology and Nova Scotia Centre on Aging, Mount Saint Vincent University, Halifax, NS, Canada. ^2^Mount Saint Vincent University, Halifax, NS, Canada. ^3^Department of Sociology, Saint Francis Xavier University, Antigonish, NS, Canada. ^4^Faculty of Health and York University Centre for Aging Research and Education, York University, Toronto, ON, Canada. ^5^Department of Geography, University of Victoria, Victoria, BC, Canada. ^6^Interior Health Authority British Columbia, Kelowna, BC, Canada. ^7^Faculty of Nursing, University of Alberta, Edmonton, AB, Canada. ^8^Institute on Aging and Lifelong Health and School of Nursing, University of Victoria, Victoria, BC, Canada. ^9^Office of the Seniors Advocate, Victoria, BC, Canada. ^10^Tefler School of Management, University of Ottawa, Ottawa, ON, Canada. ^11^Vancouver Island Health Authority, Victoria, BC, Canada. ^12^Faculty of Nursing and School of Public Health, University of Alberta, Edmonton, AB, Canada.

## Endnotes


[1] See Jull et al^9^ for a more detailed discussion of how the various research methods and approaches overlap or diverge, both in theory and practice.



[2] RAI-MDS 2.0 “is the standardized assessment tool for admission, quarterly, significant change in health status and annual assessments for each resident” in LTC.^17^



[3] Advice Seeking Networks in Long Term Care, Translating Research in Elder Care (TREC), The Canadian Health Human Resource Network, Re-Imaging Long-Term Care, Initiative for a Palliative Approach in Nursing: Evidence and Leadership (iPanel) and the Care and Construction Team [https://trecresearch.ca/research/advice_seeking_networks_in_long_term_care; https://trecresearch.ca/about/people; https://www.hhr-rhs.ca/index.php?lang=en; http://reltc.apps01.yorku.ca/our-team; https://www.ipanel.ca/#; http://www.careandconstruction.ca/].



[4] The methodological details and specific findings of each of these teams will be discussed in subsequent manucripts.



[5] These triangulation methods seek data congruence and confirmation of robust findings; however SALTY is also guided by a critical methodology that welcomes and analyzes any tensions and contradictions across findings that might be revealed throughout the research process.

